# You Must Be Joking! Benign Violations, Power Asymmetry, and Humor in a Broader Social Context

**DOI:** 10.3389/fpsyg.2019.01380

**Published:** 2019-06-19

**Authors:** Leo Kant, Elisabeth Norman

**Affiliations:** Department of Psychosocial Science, Faculty of Psychology, University of Bergen, Bergen, Norway

**Keywords:** benign violation theory, psychological distance, social distance, culture, power asymmetry, destructive leadership, anger, humor perception

## Abstract

Violated expectations can indeed be funny, as is acknowledged by incongruity theories of humor. According to the Benign Violation Theory (BVT), something is perceived as humorous when it hits the “sweet spot,” where there is not only a violation, but where the violation is also perceived as benign. The BVT specifies how psychological distance plays a central role in determining whether a certain event, joke, or other stimulus is perceived as benign or malign. In line with the aims of this research topic, we specifically address how this “sweet spot” may be influenced by *social distance*. This form of psychological distance has so far received less attention in the BVT than other forms of distance. First, we argue that the BVT needs to distinguish between *different perspectives* in a given situation, i.e., between the joke-teller and the joke-listener, and needs to account for the social distance between the two parties as well as between each of them and the joke. Second, we argue that the BVT needs to acknowledge possible *power asymmetries* between the two parties, and how asymmetries might influence the social distance between the joke-teller and joke-listener, as well as between each of these and the joke. Based on the assumption that power influences social distance, we argue that power asymmetry may explain certain disagreements over whether something is funny. Third, we suggest that *cultural differences* might influence shared perspectives on what is benign vs. malign, as well as power balance. Thus, cultural differences might have both a direct and an indirect influence on what is perceived as humorous. Finally, we discuss potential implications beyond humor, to other social situations with border zones. Close to the border, there is often disagreement concerning attempted violations of expectations and norms, and concerning their nature as benign or malign. This can for instance occur in sexual harassment, #MeToo, bullying, aggression, abusive supervision, destructive leadership, counterproductive work behavior, organizational citizenship behavior, parenting, and family relations. New understanding of border zones may thus be gained from BVT along with our proposed systematically mismatched judgments which parties could make about attempted benign violations.

To the extent that amusement can be seen as an emotion, it is perhaps the emotion for which there is the strongest uncertainty as to what type of antecedents elicit it (McGraw et al., 2014; Martin and Ford, 2018). The fundamental question that any psychological theory of humor needs to explain is why something is perceived as funny and other things are perceived as not funny. A theory developed to answer both questions is the *Benign Violation Theory* (BVT) (McGraw and Warren, 2010; McGraw et al., 2012, 2014; Warren and McGraw, 2016). According to this theory, two types of appraisals must be simultaneously present for something to be regarded as funny. First, the stimulus must represent *a violation* which is contrary to expectations and threatens the person’s view of what the world “ought” to be. Examples could range from being “attacked” by a friend trying to tickle you, to violating a linguistic norm. Second, the violation must be perceived as *benign*, which may be influenced by several factors. In the current paper, we specifically focus on the social component of psychological distance (cf. Trope and Liberman, 2010). As will be accounted for in more detail later, increased psychological distance makes minor events appear less funny, and more serious events more funny (McGraw et al., 2012).

However, the BVT has certain limitations, which constitute the starting point for this paper. One is that even jokes that include norm violations not regarded as benign can sometimes be perceived as funny (Olin, 2016). Another is the failure of the theory to account for disagreements between people as to whether something is funny within a given situation (Meyer, 2000). In our view, theories of humor also need to address why people sometimes tell jokes that others may find insulting or inappropriate. Clearly, what is intended to be funny by someone telling a joke is not always perceived as such by others. Even seemingly intelligent and emotionally sensitive people sometimes make jokes that others find offensive. For instance, a sexual joke told by a leader to a follower in a workplace, may be perceived as harassment rather than a joke. The #MeToo campaign has shown that sexual harassment often occurs in cases where someone tried (or claimed to try) to be funny. Additionally, it often occurs in relationships of asymmetric power, and may be influenced by culture (Luthar and Luthar, 2002).

We suggest that the BVT could potentially be applicable to a broader array of situations if it included three additional elements: firstly, a distinction between the *joke-teller* and *joke-listener*; secondly, the role of *power differences*; thirdly, the acknowledgement of the *cultural context* in which a joke is told. All three elements are relevant to the model’s predictions about the role of psychological distance in humor. Note that we limit our discussion to cases in which humor is used with the intention of amusing others, rather than for other communicative purposes (cf. Meyer, 2000).

## Psychological Distance in the Benign Violation Theory

According to the BVT, psychological distance reduces the tendency for people to perceive aversive stimuli or events as *threatening* (McGraw et al., 2014). When something is perceived as psychologically distant, people tend to represent them more *abstractly* (Trope and Liberman, 2010). The more psychologically distant a violation is, the more likely it therefore is to be perceived as benign. A violation can take the form of a threat to a person’s physical well-being, identity, or cultural, communicative, linguistic, and logical norms (Warren and McGraw, 2016; Warren et al., 2018). A threat is benign when perceived as “safe, harmless, acceptable, nonserious, or okay” (Warren et al., 2018, p. 5). Examples used by McGraw et al. (2012) include joking about someone stubbing their toe yesterday or being hit by a car 5 years ago. Importantly, the theory is not only concerned about what makes something funny, but also about what makes something *not* funny. A violation that is too harmless or too severe is not funny. Examples include, respectively, making a joke about someone stubbing their toe 5 years ago, or someone being hit by a car yesterday (McGraw et al., 2012).

The BVT builds on Trope and Liberman (2010, p. 440), construal level theory of *psychological distance*, in which psychological distance is defined as the “subjective experience that something is close or far away from the self, here, and now.” The theory distinguishes between four dimensions of psychological distance: firstly, *temporal distance*, i.e., whether something happened recently or a long time ago; secondly, *geographical distance*, i.e. whether something is physically near or far away; thirdly, *hypotheticality*, i.e. whether something is actually happening/perceived or only imagined; fourthly, *social distance*, which Liberman et al. (2007) exemplified as being determined by whether something happens to oneself or others, involves someone who is familiar or unfamiliar, or involves someone who belongs to an in-group or out-group. They also highlighted the relevance of social power.

## The “Sweet Spot” of Humor is Also a Matter of Social Distance

A fundamental question in the BVT is to identify the area within which something is regarded as simultaneously benign and a violation. In a longitudinal study on temporal distance and humor, McGraw et al. (2014, p. 567), “posit the existence of a sweet spot for humor—a time period in which tragedy is not too close nor too far away to be humorous.” Throughout this paper, we use the term *sweet spot* synonymously with the distance range (temporal, geographical, social, or hypothetical) at which a violation is seen as benign for a given person or a dyad, and thus being potentially funny.

One limitation to empirical studies of the BVT is that they have not addressed all forms of psychological distance to an equal extent. Even though all four forms of distance are mentioned in the BVT literature, the main focus seems to be on temporal and geographical distance (e.g., McGraw et al., 2012, 2014). Accordingly, more is known about the sweet spot of humor in relation to these two distance dimensions than about hypothetical and social distance. Importantly, we know little about how the sweet spot for humor is influenced by social factors, including whether it happens to yourself or someone else, whether that “someone” is familiar or unfamiliar to you, or belongs to an in-group or out-group. Similarly, we know little about whether and how the sweet spot for humor may be influenced by social power.

The main emphasis here is on social distance, defined as the felt distance or closeness to another person or groups of people (Stephan et al., 2011). When we address the psychological distance between a person and a joke, social distance refers to the felt distance between the focal person and the individual, group, cultural practice, norms, or roles that the joke is concerned with. In the instances where our claims refer more broadly to psychological distance, we use this broader term.

A stronger focus on the role of social distance in humor also requires that theories explicitly distinguish between *different social perspectives*. This is because the sweet spot for humor may differ between people. The existence of different perspectives is not explicitly acknowledged in BVT, which instead largely focuses on situations where there is agreement over whether something is funny or not.

Notably, Kim and Plester (2019) also addressed the social element of humor, including the existence of multiple social roles and perspectives. They demonstrated how the perception and usage of humor in an organizational setting may be influenced both by the persons’ relative social positions and the culture at large.

## The Importance of Social Context, Power, and Culture

Olin (2016) pointed to questions that theories of humor need to explain, over and above the fundamental question of what makes something funny or not funny. The majority of these questions related to the social/societal context in which humor takes place. The importance of knowing more about the social context of humor is also implicated in the current research topic. This goes both for the organizational context (Kim and Plester, 2019) and for the larger societal context (e.g., Jiang et al., 2019).

In the BVT, the sweet spot of humor has to do with identifying something which is a violation of the expected, while simultaneously being benign. However, to the extent that a humorous situation involves multiple persons, the sweet spot would also be likely to depend on social variables. One example is *roles*. You can play around with roles—violate them—in a benign fashion. For example, a violation can occur when a person by telling a joke steps out of their expected role. Such violations may be funny, for instance when a teacher starts dancing on the table. However, there are also potentially adverse sides of breaking roles or creating ambiguity around them (e.g., Örtqvist and Wincent, 2006; Eatough et al., 2011). One example would be a general practitioner who jokes with a patient about breaking doctor-patient confidentiality.

Violation of *social expectations* may also be funny. Social expectations may pertain to roles, but also to the activities, tasks, and goals that social relationships involve. Our social relationships to family, friends, leaders, and coworkers can involve a goal of catching a bus, getting a work task done, or getting the children to bed at night. It is therefore possible to violate the relationship itself, but also the activities or organizational interests (cf. House and Javidan, 2004; Einarsen et al., 2007).

In line with this general focus on the social element of humor, Olin (2016) differentiated between the *joke-teller* and *joke-listener.* This distinction is drawn in Olin’s discussion of jokes that implicate negative group stereotypes. Here, attitudes and beliefs of different parties may influence the extent to which a joke is perceived as humorous or harmful.

Interestingly, Kim and Plester (2019) drew similar distinctions in an ethnographic study of the influence of roles and hierarchy on humor perception and expression in Korean work settings. They found substantial differences in the contents of and reactions to humor among subordinates and superiors. For low-power individuals, humor expressions even had negative emotional consequences. This study demonstrates the importance of addressing multiple social perspectives and power differences in humor research.

Our theoretical account is also in line with a recent empirical study by Knegtmans et al. (2018), who studied the influence of power on the perception of jokes. However, here power was conceptualized as a temporary *psychological state*. In contrast, our conceptualization of power goes beyond temporary states, feelings or experiences of power. We focus on more stable power asymmetries deriving from hierarchical differences in organizations, and from social roles. Examples include a leader’s position compared to a subordinate’s, an emperor’s compared to a peasant’s, and a parent’s compared to a child’s. Furthermore, we emphasize the important role of *culture*, which is likely to have a direct influence on the shared norms for what constitutes a violation and what is considered benign (e.g., Gray and Ford, 2013). Culture could also influence norms for expressing amusement. It might also influence power differences and social distance in various ways. Thus, it could have both direct and indirect effects on humor perception.

To the extent that humor perception is influenced by power differences and culture, this may largely take place through their influence on social distance. Even though social distance, power, and culture are discussed separately in subsequent sections, it is important to keep their interrelatedness in mind.

## Three Suggested Elements that Could be Added to Benign Violation Theory

We now turn to three components that in our view need to be included in the BVT to increase its explanatory value. These are in line with Olin’s (2016) suggestion to focus on the social aspects of humor in understanding when incongruent events are perceived as humorous and when they are not. They specifically address “boundary areas” of humor (e.g., Plester, 2016). These components are (1) distinguishing between the joke-teller and the joke-listener; (2) addressing possible power differences between the joke-teller and the joke-listener; and (3) acknowledging the influence of culture on the relationship between power differences and humor.

Note that this discussion will be limited to situations in which someone *intentionally* tells a joke to someone else, and where the intention is to be funny by hitting the sweet spot of both joke-teller and joke-listener. This is in contrast to any intentionally dark uses of humor (cf. Plester, 2016) aimed beyond the sweet spot, deliberately hurting the joke-listener, such as in power play, conflicts, ostracism, or bullying. A joke-teller may attempt both to split a crowd, hit the sweet spot with someone, while victimizing others (cf. Salmivalli, 2010). Again, our discussion concerns attempts to hit the sweet spot, and associated risks of over- or undershooting.

### Joke-Teller vs. Joke-Listener

Empirical research on the BVT seems to mostly address situations in which *someone* regards or does not regard *something* as funny (McGraw and Warren, 2010; McGraw et al., 2012, 2014; Warren et al., 2018). However, one does not specifically differentiate between a joke-teller and a joke-listener, and whether different perspectives may influence the extent to which something is perceived as benign, a violation, and funny. If one is to understand humor at a level beyond the individual, this distinction is essential.

Thus, psychological distance in the BVT seems to normally be conceived of in terms of the distance from the *person* (who could either be the joke-teller or the joke-listener) to the *something* (the stimulus, which could either be a joke or an episode). The social setting in which the *something* is observed, heard, or experienced is not taken into consideration. In reality, a social setting would normally involve several people who would have different roles and perspectives and could in principle disagree as to whether the joke was a violation, whether it was benign, and whether it was funny. We choose here to use Olin’s (2016) terminology of *joke-teller* and *joke-listener*. Other related concepts are *humor user*, *target person*, and *audience* (Meyer, 2000).

Whether a joke told by a joke-teller to a joke-listener is perceived as funny by either or both of them could depend on a number of factors that would influence the extent to which something would simultaneously be seen as benign and a violation. It can be meaningful to analyze this in terms of the following four subtypes of *social distance* in a joke setting, namely sections “Social Distance Between Joke-Listener and Joke”; “Social Distance Between Joke-Teller and Joke”; “Social Distance Between Joke-Teller and Joke-Listener”; and “The Relative Social Distance Between Joke-Teller, Joke, and Joke-Listener.” We think that all four forms of relationships are relevant for both parties. However, because the joke-teller is the active part, s/he is perhaps more likely to actively consider these distances when preparing for a joke delivery than the joke-listener is when hearing a joke. In the following, we only provide selected examples illustrating either of these perspectives.

#### Social Distance Between Joke-Listener and Joke

The one form of distance that McGraw et al. (2012, 2014) have most clearly addressed is the psychological distance between the joke-listener and the joke. They addressed how a joke-listener can feel temporally close or distant to an event, depending on whether it happened recently or long ago. Similarly, a joke can pertain to something geographically close or far away. Here, we argue that a joke-listener and a joke also may be *socially distant* or *socially close*, as perceived by the joke-listener or joke-teller.

The social distance to a joke would be conceptualized slightly differently depending on whether or not the joke directly addresses specific people. To the extent that a joke refers to a person or group of people, the social distance to the joke would directly correspond to the social distance to those involved, whether it was a specific person or a group. Even jokes that do not refer to specific people may still have contents that are relevant to the social roles, social identities, attitudes, cultural practices, values, and norms of a joke-listener. The social distance to the joke would then depend on the person’s commitment or dedication to each of these. For instance, Hemmasi et al. (1994) showed that sexist jokes targeting the opposite sex were regarded as more funny (by men and women) than sexist jokes targeting one’s own gender. Similarly, violations could be more likely to be viewed as benign if concerned with an out-group or unfamiliar persons.

The social and ethnic groups and cultures to which the joke-listener belongs or associates her-/himself with would obviously be important. The history and identity of that larger group or culture in general could also be relevant.

#### Social Distance Between Joke-Teller and Joke

Another form of distance seemingly overlooked by the BVT is the perceived/attributed social distance between the joke-teller and joke, as perceived by either party. This refers to whether the joke-teller is perceived as either *socially distant from* or *socially close to* the content of the joke. The joke-teller’s perception of this may be likely to influence what s/he chooses to joke about. It is well established in research on attribution that emotional responses are highly influenced by inferences of responsibility, including intent, causal controllability, free will, and other associated concepts (e.g., Weiner, 1993, 2006). Therefore, the joke-listener’s perception of this form of distance could influence how s/he perceives the *intention* of the joke-teller. For instance, imagine someone (with intact vision) who tells a joke about blind persons. Whether this violation is seen as benign, and whether the joke is perceived as funny, might depend on whether the joke-perceiver knows or thinks that the joke-teller has had a close personal relationship with someone who is blind.

Thus, the perceived social distance between the joke-teller and the joke might be influenced by the one person’s perception of the other’s attitudes, social roles, social identities, cultural affiliation, etc. (Liberman et al., 2007; Trope and Liberman, 2010). The perception of the joke-teller’s actual roles and identities may be more or less accurate.

#### Social Distance Between Joke-Teller and Joke-Listener

The social distance between the joke-teller and joke-listener is also relevant. This point is related to but not overlapping with the two previous points.

The closeness of the relationship between the two parties is important. If the joke-teller and joke-listener do not have a close personal relationship, it is relevant whether the joker is familiar or unfamiliar, or belongs to an in-group or an out-group. Note that the two parties may have a different idea of what the social distance is between them.

*Power differences*, that is the relative power between two parties, appears to be a crucially important variable in this context. Hemmasi et al. (1994) asked survey respondents to indicate how likely they would be to perceive sexual and sexist humor as sexual harassment, if coming from a person of the opposite gender who was either a coworker or leader. Both sexist and sexual gender-related jokes were more likely to be perceived as sexual harassment when the joke-teller was a leader rather than a coworker. Hemmasi et al. (1994, p. 1125) concluded that “Regardless of the manager’s intent (i.e., to deliberately insult/intimidate the subordinate, or merely to innocently retell an ‘amusing’ joke), such behavior is a high-risk activity.” We will discuss power differences in section “Power Differences and the Case of Asymmetry.”

#### The Relative Social Distance Between Joke-Teller, Joke, and Joke-Listener

Importantly, any of the three previous types of social distance cannot be understood in isolation. Whether a joke is perceived as a benign violation will also depend on the relative distances between the joke-teller, joke, and joke-listener. The social distance between a joke-listener and joke-teller may moderate whether a joke is perceived as benign or not. For instance, a sexist joke about women, told to a woman by a man unknown to her, and belonging to a different social or cultural group, could be perceived as more malign and offensive, and less funny, than the same joke told by a close female colleague belonging to one’s in-group. Similarly, imagine your grandfather attempting a joke, using a term which is insulting among millennials. If you attribute a well-meaning intent and infer it to be unknowingly done due to distance to the lingo of the youth, you may still laugh. Thus, we suggest the relative distance between joke-teller, joke, and joke-listener as a fourth type of social distance relevant to humor. Again, different parties may disagree in their perception of these relationships in a given situation.

#### Implications

Note that all four types of distance identified here (sections “Social Distance Between Joke-Listener and Joke” to “The Relative Social Distance Between Joke-Teller, Joke, and Joke-Listener”) could also be applied to other dimensions of psychological distance. For instance, the joke-teller and joke-listener could be temporally or geographically close or far apart, as could the content of the joke be to either or both parties. However, since the focus of this paper is on the social dimension, we will not discuss the influence of the other dimensions any further. Nevertheless, it is important to keep in mind that social distance may be influenced also by geographical and temporal distance. For example, a leader who sits in the office next door and who you interact with frequently might (from your perspective) feel socially closer than a leader who sits in the headquarters in a different city, and who you only communicate with by email a few times every month (cf. Antonakis and Atwater, 2002).

### Power Differences and the Case of Asymmetry

#### Asymmetric Power and Social Distance

In our view, a potentially important element in the relationship between the joke-teller and the joke-listener is that of *power*. Notably, the theory of psychological distance that the BVT largely draws on has specified that *power* is a predictor of social distance. The presence of power differences between individuals or groups of individuals may influence the perceived social distance of both parties. High-power people see themselves as more different and distant from others than low-power people do (Liberman et al., 2007). This is of course primarily a question of *relative* distance. Power differences could very well increase the *absolute* social distance as perceived by the low-power individual—a notion compatible with theories on leader distance (e.g., Antonakis and Atwater, 2002). The important point is that power differences would always increase the social distance as perceived by the high-power individual even more.

In addition, Smith and Trope (2006) argued that increased power leads to increased tendencies to think more abstractly, a tendency indicative of larger social distance from others. They conducted a series of experiments where participants were primed with power concepts, and claimed to find that such priming increased people’s tendency for abstract, high-level construals.

If power is an additional determinant of construal level, power differences may be relevant in the search for a “sweet spot” within which both joke-teller and joke-listener can agree on a joke constituting a benign violation. It is therefore surprising that these elements have not yet been systematically integrated into the BVT.

In their *Social Distance Theory of Power* (SDTP), Magee and Smith (2013) have built on the positive correspondence between power, abstract construals, and increased social distance reported by Smith and Trope (2006). A central point in SDTP is that power asymmetry may lead to asymmetry in the perceived social distance between two parties of a dyad: whereas a low-power individual may feel relatively close to a high-power individual, the high-power individual may feel relatively distant to the same low-power individual. Their theory is mostly concerned with dyadic relationships where power is related to interdependence. However, it could be relevant to other types of relationships where interdependence is less present or central than in dyads.

Of particular interest are those cases where there is *asymmetric power* between the joke-teller and the joke-listener. What Magee and Smith (2013) hypothesized about the relationship between asymmetric experiences of social distance and power could provide an important contribution here.

#### Asymmetric Power and the Benign Violation Theory

How can the basic ideas in the SDTP (Magee and Smith, 2013) be incorporated into the BVT? In principle, asymmetric power might influence all four forms of social distance presented previously.

Most fundamentally, power asymmetry might influence the *social distance* between a joke-teller and a joke-listener. According to Magee and Smith (2013), this in turn may have several cognitive and emotional consequences for how the other person is perceived. For example, they argue that high power is associated with a reduced feeling of being similar to the other person. In contrast, low power is characterized by a stronger tendency to feel similar when comparing oneself to others. Moreover, high power is associated with reduced attention and responsiveness to the mental states, thoughts and feelings of other people. According to the SDTP, this may lead high-power individuals in an asymmetric relationship to display *empathic inaccuracy* (Magee and Smith, 2013). This is consistent with experimental findings showing that high social class predicts increased unethical behavior (Piff et al., 2012): the unethical behaviors in the experiments included ignoring shared norms, even rules, with high-social class individuals allowing themselves to break traffic rules and steal candy from children. It is worth noting that the mediating mechanism was a baseline-difference in mind-set between high- and low-class individuals.

How does this influence whether something is perceived as a benign violation, and funny, in a situation where a joke-teller tells a joke to a joke-listener? According to predictions derived from the SDTP, this would crucially depend both on which form of social distance (sections “Social Distance Between Joke-Listener and Joke” to “The Relative Social Distance Between Joke-Teller, Joke, and Joke-Listener”) we are concerned with, in combination with the particular power balance in the relationship.

Let us first turn to the case where the joke-teller is in the high-power position, and the joke-listener is in a low-power position. This is a potentially risky situation in the sense that the joke-teller experiences a greater social distance both toward the joke and the joke-listener than the joke-listener does. As a consequence, it takes more for the high-power joke-teller to regard something as a violation, and more for something to be perceived as benign. For instance, the joke-teller may feel that it is more appropriate to make jokes about events that are closer in time, geographically, or socially, than the joke-listener feels. Another way to put it—the *impropriety threshold* (for when a violation is no longer perceived as benign) is higher for the high-position joke-teller (cf. Geddes and Callister, 2007). This might not pose a problem in cases where the power distribution is symmetrical. However, when the joke-listener is in a low-power position, their impropriety threshold becomes correspondingly lower. This might imply a smaller (or no) overlap between the sweet spots of the two parties. Thus, a violation could more easily be perceived as malign. If the high-power joke-teller is also less “empathically accurate” (cf. Magee and Smith, 2013), s/he might not realize that the violation was perceived as malign by the other, which could contribute to a vicious cycle.

Magee and Smith (2013) also claimed that power is related to the tendency to experience socially engaging versus disengaging emotions. They argued that high-power individuals are less motivated to affiliate with others and therefore less likely to experience socially engaging emotions and more likely to experience socially disengaging emotions. To the extent that humor is a socially engaging emotion, an additional prediction can therefore be that this tendency further increases the high-power joke-teller’s threshold for experiencing something as funny. This could further increase the risk of offensive jokes.

As the idiom goes, it is lonely at the top. It is also safer to shout out. Those below may however perceive the same as a beginning avalanche. For a high-power individual to hit the sweet spot with a joke to a low-power individual, s/he needs to decrease the severity or increase the social distance between the joke content and the joke-listener. This principle is perhaps reflected in the frequent practice of making jokes about people from a neighboring country. For instance, Swedes among themselves joking about Norwegians and vice versa, and Americans joking about Canadians.

What then about the case of a joke-teller being in a low-power position and the joke-listener in a high-power position? This should be a less critical situation. Here, it would take more for the high-power joke-listener to perceive something as a violation, and to perceive a violation as malign, than it would take for the low-power joke-teller. Thus, the biggest danger might perhaps be that the high-power joke-listener would be less likely to be amused by jokes that the low-power joke-teller thinks represent benign violations. This is indeed consistent with what Knegtmans et al. (2018) found when they induced experimental participants with states of high or low power. High-power participants were less likely to rate jokes as inappropriate, offensive, and also less funny. These findings are compatible with the assumption that high-power individuals’ “impropriety threshold” (cf. Geddes and Callister, 2007) was higher, and that they may not have perceived the joke as a violation. Thus, asymmetric power relation is also likely to involve an asymmetry in what a joke-teller and a joke-listener regards as funny, offensive, or simply boring. Again, the challenge would be to find the sweet spot that overlaps for the joke-teller and joke-listener. For a low-power individual to hit the sweet spot with a joke to a high-power individual, one needs to increase the severity or to somehow decrease the distance, e.g., getting more personal with the high-power individual. The latter may of course have cultural limitations/restrictions, or even involve cultural taboos—one is not always at liberty to inform the emperor that he is in fact naked.

We do not claim to be the first to suggest that social power may be an important variable for the BVT to take into account. Knegtmans et al. (2018) also addressed possible implications for the jokes one might choose to tell. However, their main emphasis was on how the power of the *joke-listener* influenced perceived inappropriateness, offensiveness, and funniness of jokes. Moreover, they did not discuss the case of asymmetric power, or possible consequences of power differences between a joke-teller and joke-listener. Additionally, their emphasis was on power as a *state variable* rather than more stable power differences.

#### Superimposed Sweet Spots?

It follows that a joke-teller in a certain power position may have one sweet spot as defined by the BVT, whereas a joke-listener in a different power position may have a different sweet spot as defined by the BVT. If one were to superimpose one over the other, it may become logical why a given joke may be offending for one person, bland for another, or if all goes well—funny for both. If the superimposition revealed nonoverlapping areas, these could be described as the “asymmetric upper” (i.e., the joke-teller considers it a benign violation, but the joke-listener considers it a malign violation—*offensive*) and the “asymmetric lower” (i.e., the joke-teller considers it a benign violation, but the joke-listener considers it benign, but not a violation—*bland*). This can be understood in relationship to section “The Relative Social Distance Between Joke-Teller, Joke, and Joke-listener.” The relative social distances involved in the triad of the joke-teller, the joke-listener, and the joke might be perceived differently by the joke-teller and the joke-listener.

### Cultural Differences

We started by discussing the different roles of the joke-listener and joke-teller. We argued that the social distance between each of these and the joke, as well as the relative distance between the three, is not always identical. This may in turn lead to differences in perception. We then turned to how power asymmetry may generate asymmetry in social distance, making it possible for the two parties to have different sweet spots of humor for a given joke in a given setting. We will now place these factors in the broader context, by highlighting three ways in which culture may influence the sweet spot of humor.

First, cultural differences may influence *the absolute level* of what is considered benign or malign for entire societies or organizations. Even though humor is a universal phenomenon, there are also cultural differences. These may concern both how humor is perceived, valued, and used (cf. Jiang et al., 2019 for a review). Jiang et al. (2019) largely focused on the broader cultural differences, especially those between Eastern and Western societies. However, differences between subcultures, i.e., between different cultural groups in a country as well as regional groups within a country, could also have an influence on humor perception and usage. The role of subcultures is illustrated by an empirical study by Gray and Ford (2013). People’s interpretation of sexist jokes differed depending on whether jokes were told in a setting where such jokes were tolerated (i.e., a comedy club) or prohibited (i.e., a workplace). Here, it may also be meaningful to point to the possible influence of organizational culture (e.g., Geddes and Callister, 2007), which could influence the absolute level of how certain groups of individuals may perceive or use humor. Cultural differences (between societies, organizations, or even families) may also influence the extent to which individuals are expected or allowed to express certain emotions. In the case of humor, this is relevant to the extent that such cultural differences concern the *appropriateness of expressing amusement* (e.g., Gottman et al., 1996; Magee and Smith, 2013). Cultures may thus dictate a shared impropriety threshold (for when a violation is no longer seen as benign). It might also be meaningful to think of cultural values influencing the permeability of the border, as well as the willingness to explore border areas. This goes for societies in general (Gelfand et al., 2011; Plester, 2016), organizations (Plester, 2009), as well as for other social entities.

Second, cultural differences may influence *power differences* in multiple ways. According to the classic theory of Hofstede (1980), high versus low power distance is one of four dimensions along which national cultures differ. Obviously, culture may therefore influence *high-power and low-power* positions as is also known from cross-cultural leadership research (e.g., Antonakis and Atwater, 2002; Chhokar et al., 2007; Aktas et al., 2015). Magee and Smith (2013) pointed to two important ways in which culture may influence power asymmetry, which in our view may be particularly relevant to the case of humor. Their first point is that since culture may influence people’s beliefs about what behavior is considered appropriate for a high-power individual, power differences may not necessarily lead to asymmetric social distance in all cultures. Their second point is that cultures may differ in the extent to which they take for granted or justify power differences. Therefore, in some cultures, low-power individuals may experience equal levels of social distance as high-power individuals in a given relationship. The implication of our current arguments is that culture could influence the circumstances under which a joke told between two individuals belonging to the same culture is seen as funny or malignant.

Our third point concerns those cases in which the joke-teller and joke-listener have different cultural backgrounds. In this case, cultural differences may influence *the relative thresholds* for each party. Using the same analogy as previously, cultural differences may cause the superimposed sweet spots to change in relative location, and perhaps even in shape.

As mentioned earlier, the important role of culture in influencing power asymmetry has to date been overlooked in studies that address the possible role of social power in BVT (Knegtmans et al., 2018). It could also be added that the influence of culture is likely to be slow to change. It is probably slower than group-level changes in hierarchical roles in an organization, or even in a family. Moreover, definitely slower than an individual level state of power (e.g., Knegtmans et al., 2018). An important message of the current paper is that the BVT needs to acknowledge how culture might influence the mechanisms specified by the theory.

## Implications of Our Claims

“*From a distance there is harmony*” *(Julie Gold)*

We have suggested that the humor mechanism accounted for by the BVT needs to be specified and extended, also beyond recent efforts (e.g., Knegtmans et al., 2018). The BVT explains why some attempts may succeed, some may fall short, and others may overshoot the sweet spot. Our emphasis on the role of potential power asymmetry may explain why a joke-teller and a joke-listener may perceive the sweet spot to be of different size and different location. Power asymmetry entails distance asymmetry, and therefore different sweet spots. This may lead to some humorous attempts to remain unnoticed by high-power individuals, and other efforts being perceived as offensive by low-power individuals. The former may involve frustrated low-power individuals not gaining acknowledgement from high-power listeners. The latter may however touch quite sinister topics, such as sexual harassment, bullying, abusive supervision, destructive leadership, and so on.

Our theoretical suggestions may have consequences for who can joke about what with whom. Do you come from a position of power, be it formal or informal? Leaders, parents, representatives of the dominant cultural group, the dominant gender, the in-group, the seniors at the workplace may all see a different sweet spot than their counterparts. This may be of value in humor research. For instance, when investigating jokes in romantic relationships, in workplaces, on the sports field, and so on.

Our small addendum to BVT is to acknowledge two aspects. Both are in line with recommendations to attend more to social contexts in humor research (Olin, 2016), and social power beyond temporary experimental states (Knegtmans et al., 2018): first, the importance of two main parties, the joke-teller and the joke-listener; second, power-related asymmetry in the cases it exists, and how it may influence four forms of perceived social distance asymmetrically. Herein lies the systematic potential for mismatched maps. When superimposing the different maps of the high-power party and the low-power party, it does not only reveal a fixed border zone, but a disputed no-man’s-land with split opinions, perhaps even a frontier for change.

Benign violations reside between two outer areas which the majority can agree on. One outer area being unequivocal good, in humor constituting the benign but non-funny. The other being the unequivocal bad, in humor the harmful where only the violation remains. In between lies the sweet spot—a violation also perceived as benign. Such sweet spots, we suggest, exist in other models of social interaction. Therefore, it could be possible to bring the BVT into a greater social context. If benign violations may take the form of any type of behavior occurring in the narrow border areas between the acceptable and unacceptable in everyday social interaction, the theoretical implications of our arguments may be broad.

Even though this paper is a conceptual analysis, we here briefly exemplify some ideas for empirical research that could be used to test our claims. The ideal way to test our model would be a full factorial design testing the joint effect of distance, power, and culture on perceived severity and amusement, inspired by existing procedures (e.g., Hemmasi et al., 1994; Knegtmans et al., 2018). However, quasi-experimental investigations could also be used. One example is to study sub-components of the model where naturally occurring power differences are relatively stable, as in hierarchical organizations such as hospital wards or families, or in organizations where hierarchies may change across time (Breevaart et al., 2014).

## Bringing Benign Violation Theory into a Broader Social Context

We now turn to other forms of benign versus malign violations, beyond humor. If the phenomena include a sweet spot as well as power differences, the BVT with our addendums may supplement the understanding of border areas in other models.

There are solemn issues in everyday life, described by established theoretical models, which also concern what can be seen as dual thresholds in social interaction. These are cases where there is a sweet spot or zone between the expected and the unexpected, the in-role behavior and extra-role behavior, the normal and the non-normal, the constructive and the destructive, the expressed and the improper, the good and the bad. Here, a benign violation would not necessarily be associated with humor or amusement, but could be associated with other positive emotions (e.g., appreciation, enthusiasm, respect) and have other positive personal and interpersonal consequences (e.g., organizational improvement, loyalty, identification).

Among the areas which we thus suggest may encompass benign violations, we find the sweet spots described more or less explicitly in relevant theoretical models. Some models clearly establish a sweet spot, whereas others only indirectly imply its existence.

An explicit sweet spot can be found in *the dual threshold model of anger in organizati*ons (Geddes and Callister, 2007), which directly corresponds to the basic notion in BVT. Not expressing anger is in the normal or unequivocally good zone. Anger above the *expressed threshold* but below the *impropriety threshold* is in the sweet spot. Expressed anger can thus quickly enter into the bad and vast realm of over-the-line aggression. A notable similarity to our line of reasoning is that Geddes and Callister (2007) argued that culture may influence where the shared thresholds are set, in their case through an implicit agreement for each organization. A dissimilarity is that our reasoning on power asymmetry opens up for multiple, simultaneous, and asymmetric fields for individuals.

Other models also attend to a form of sweet spot, although the correspondence to BVT mechanisms is less explicit. For instance, *organizational citizenship behavior* (OCB) (cf. Spector and Fox, 2010) refers to a form of extra-role behavior, where an employee goes above and beyond the call of duty. It is thus a violation of the expected or contractual obligations, which also is deemed benign. However, discussions on OCB include reflections on the facts that (1) the expected behavior should not be left undone and (2) everyone cannot exclusively perform out of the ordinary OCB. The bland, boring, and necessary task must be done—someone must sweep the floor. In other words, there is a “too much” in OCB, which may become offensive. This behavior can thus be both benign and malign if present. In a similar vein, in a study on *workplace bullying* enacted by leaders, Rayner and Cooper (2003) discussed *spectrum behavior*. That is, behavior which if present could be either benign or malign. Humor with its bright and dark uses could in general be considered a type of spectrum behavior (cf. Plester, 2016). This is in contrast to behavior which, if present, is *either* exclusively malign or benign. Examples are, respectively, humiliating people, or displaying constructive leader behavior (Rayner and Cooper, 2003).

Yet other models give a lot of attention to the good or the bad, but less to the border area. An example is *destructive leadership* (Einarsen et al., 2007) pertaining to leaders—along with several alternate concepts such as *abusive supervision* (Tepper, 2000) or *toxic leadership* (Padilla et al., 2007), and *counterproductive work behavior* (Spector and Fox, 2010) pertaining to subordinates. In these models, it is clear that severe anti-subordinate/interpersonal and anti-organizational behavior is bad, and correspondingly, that strongly pro-subordinate/interpersonal and pro-organizational behavior is good. The border may however be ambiguous, hard to define, and influenced by a variety of factors.

Some of these solemn issues by definition involve power asymmetry, for instance leaders and subordinates operating in a formal hierarchical system, where anger, destructive leader behavior, and destructive subordinate behavior occur. However, such behaviors may also take place in other contexts of power asymmetry, for example in families. Gender-related asymmetry may for instance be found in matriarchies and patriarchies. In any culture, societal or subcommunity, there are also dominant in-groups and minority out-groups with power asymmetries aplenty.

A methodological implication is the need to consider appropriate levels of analysis (Yammarino and Dansereau, 2008). By this, we mean that the individualized or dyadic level of analysis may be particularly relevant in the border zone, and group-level analysis more relevant with increasing levels of severity (be it good or bad). That is, in the border zone, relative power and distance will lead to individual variation, which may be detectable as dyadic level significant effects. However, with such variation, an entire team or an entire family or audience may not agree on the ratings. Thus, inter-rater reliability should be low. With increasing severity, more individuals will agree on the joke being bland or offensive, the expressed anger being improper, or the leadership behavior being clearly destructive. High social distance is notably also associated with group-level outcomes (Antonakis and Atwater, 2002).

A potential theoretical implication is whether change is possible through the suggested mechanisms. In the “sweet spot” lies the potential for positive change. Humor used with ambitions to “develop organizational culture” has been empirically reported (Plester, 2016, p. 88). We may consider an appropriate level of OCB as a case of benign violations. We may also consider whether nonviolent change such as that of Gandhi could be another. Gandhi (1940) emphasized just barely breaking the (oppressive) law, without hurting others, and while telling the truth. In everyday working life, leaders may need to violate the interests of either the organization or the subordinates at times, in order to facilitate change. An example of the former would be a middle manager motivated by a wish to protect the flock while breaking organizational interests, thus displaying *friendly-disloyal* leader behavior (cf. Einarsen et al., 2007). Examples of the latter would include *virtuous betrayal* toward subordinates which Krantz (2006, p. 221), argues leaders sometimes have to do “in the service of higher purposes.” As this involves pushing subordinates beyond their comfort zone, it bears similarities to borderline *tyrannical* leadership (cf. Einarsen et al., 2007).

In contrast, a change for the worse is often diffuse and done in a series of malign violations, each of which could be minor, i.e., just passing the impropriety threshold. Corrupt organizations or totalitarian states are rarely created overnight. Passivity and silence are often required of many, as in the rise of the Nazi regime (Lewin, 1943) or with the #MeToo. Malign violations are accepted in spite of opposing views. Power asymmetry could be an integral part, where the low-power person, the new employee, the young, and so on want to appeal to the high-power person. Perhaps they modify their emotions for organizational survival as they typically do, suppressing negative emotions and exaggerating positive emotions (Glasø et al., 2006), including laughing at the rich man’s joke. The stepwise nature of malign violations might increase the likelihood for change. The high-power individual takes an ever so little step over the line, “and then, if you are drawn in, next week it will be something a little further from the rules” (Lewis, 1949, p. 63). With the behavioral step already taken, the low-power individual is left only with the opportunity to change the values in order to resolve the *cognitive dissonance* (Festinger, 1962).

We could also mention other social phenomena characterized by sweet spots of acceptable behavior, and where the border between the benign/acceptable and the malign is likely to be influenced by social distance, cultural norms, and power distribution. Examples would include (but not be limited to) white lies, courtesy, and cursing.

## Concluding Remarks

Our attempt to specify the role of social distance in the BVT, focusing on power differences and culture, could be seen as a first step in identifying the mechanisms that are involved when social norms and expectations are unsuccessfully violated. Even though this paper has focused on the *intentional* joke leading to *unintentional* crossings, inappropriate crossings may of course also be done intentionally (cf. Plester, 2016).

We have focused on voluntary behavior, intended on hitting the funny—the sweet spot—which is both a violation of the expected and something benign. We have argued that there may exist a systematic tendency explaining certain cases of mismatch between parties, with a potential for transgressions. This systematic tendency cannot be fully understood unless set in a social context where the potentially great influence of culture and power asymmetries are incorporated. This has implications for our understanding of humor in general, humor in asymmetric power relationships, as well as for understanding other situations of benign violations, far beyond humor.

## Author Contributions

LK and EN have contributed in all parts of the research process, including developing the conceptual framework, writing and revising the manuscript. LK had the main responsibility for conceptualizing and writing those parts of the manuscript that address implications and applications of the framework in a broader context.

### Conflict of Interest Statement

The authors declare that the research was conducted in the absence of any commercial or financial relationships that could be construed as a potential conflict of interest.
